# A novel SARS-CoV-2 Beta RBD DNA vaccine directly targeted to antigen-presenting cells induces strong humoral and T cell responses

**DOI:** 10.1038/s41598-023-46223-8

**Published:** 2023-11-02

**Authors:** Katarzyna Kuczkowska, Louise Bjerkan, Elisabeth Stubsrud, Hannah Cuthbertson Husbyn, Stalin Chellappa, Anette Hauge, Renate Skarshaug, Maria Lyngaas Torgersen, Joel Benjamin Heim, Marthe Jøntvedt Jørgensen, Christian Winther Wold, Mariane Høgsbjerg Schleimann, Martin Tolstrup, Stine Granum, Agnete Brunsvik Fredriksen, Mikkel Wandahl Pedersen, Gunnstein Norheim

**Affiliations:** 1 Nykode Therapeutics AS, Oslo Research Park, Gaustadalléen 21, 0349 Oslo, Norway; 2 Veterinærinstituttet, Elizabeth Stephansens Vei 1, 1433 Ås, Norway; 3 Department of Clinical Medicine/Infectious Diseases, Aarhus University, Palle Juul-Jensens Boulevard 45, 8200 Aarhus N, Denmark

**Keywords:** Vaccines, DNA vaccines

## Abstract

Throughout the COVID-19 pandemic, several variants of concern (VoC) of the severe acute respiratory syndrome coronavirus 2 (SARS-CoV-2) have evolved, affecting the efficacy of the approved COVID-19 vaccines. To address the need for vaccines that induce strong and persistent cross-reactive neutralizing antibodies and T cell responses, we developed a prophylactic SARS-CoV-2 vaccine candidate based on our easily and rapidly adaptable plasmid DNA vaccine platform. The vaccine candidate, referred to here as VB2129, encodes a protein homodimer consisting of the receptor binding domain (RBD) from lineage B.1.351 (Beta) of SARS-CoV-2, a VoC with a severe immune profile, linked to a targeting unit (human LD78β/CCL3L1) that binds chemokine receptors on antigen-presenting cells (APCs) and a dimerization unit (derived from the hinge and C_H_3 exons of human IgG3). Immunogenicity studies in mice demonstrated that the APC-targeted vaccine induced strong antibody responses to both homologous Beta RBD and heterologous RBDs derived from Wuhan, Alpha, Gamma, Delta, and Omicron BA.1 variants, as well as cross-neutralizing antibodies against these VoC. Overall, preclinical data justify the exploration of VB2129 as a potential booster vaccine that induces broader antibody- and T cell-based protection against current and future SARS-CoV-2 VoC.

## Introduction

The initial COVID-19 pandemic outbreak began in December 2019, and since then, the causative virus, severe respiratory syndrome coronavirus 2 (SARS-CoV-2), has evolved, resulting in the emergence of many new variants worldwide. Some of these have been defined as variants of concern (VoC) and shown to be associated with one or more of the following changes of global health significance: (i) increase in transmissibility or detrimental change in COVID-19 epidemiology; (ii) increase in virulence or change in clinical disease presentation; (iii) decrease in the effectiveness of public health and social measures or available diagnostics, vaccines or therapeutics^1^ (www.who.int). The list of VoC has included Alpha B.1.1.7, Beta 1.351, Gamma P.1, Delta B.1.617.2, Omicron B.1.1.529, and other Omicron lineages.

The major challenge faced by COVID-19 vaccine developers is to develop vaccines that can prevent infection and severe disease while addressing the rapidly emerging new VoC that evades vaccine-induced spike-neutralizing antibodies. A successful vaccine could prospectively exploit cross-reactive T cell responses that are less susceptible to immune escape^2^, along with the induction of broad, strong, and protective neutralizing antibodies. Here, we developed a prophylactic SARS-CoV-2 vaccine candidate, referred to as VB2129, based on a clinically validated DNA plasmid vaccine platform. The plasmid DNA encodes a chimeric protein designed to enhance antigen uptake by targeting antigen-presenting cells (APC). VB2129 encodes homodimeric vaccine proteins in which each chain comprises a targeting unit, a dimerization unit derived from the hinge and C_H_3 exons of human IgG3, and an antigenic unit^3, 4^. Based on results with prior candidate^5^, CCL3L1 (LD78β) was selected as the targeting unit because of its ability to attract APCs and drive antigen uptake through chemokine receptors CCR1 (C–C motif chemokine receptor 1) and CCR5 (C–C chemokine receptor type 5)^4^, for effective presentation of antigenic epitopes on MHC class I or II molecules and activation of cytotoxic CD8^+^ and CD4^+^ T-helper cells. CCL3L1 binds to CCR1 and CCR5 receptors on the critical APC subset cDC2 but binding is not limited to this subset^6^. The hinge region facilitates dimerization through disulfide bonds, while C_H_3 contributes further to effective dimerization through hydrophobic interactions.

In addition to increased antigen uptake through receptors on APC for presentation and activation of T cells, the antigen bivalency of targeted homodimeric vaccines enhances B cell responses^7–9^. Bivalent display of antigens is shown to cross-link B cell receptors (BCR) and using fusion with the targeting unit, promote synapse formation between APCs and B cells, facilitating early B cell signaling and enhancing effective antibody responses^10, 11^.

The plasmid DNA vaccine platform is a safe technology with an intrinsic adjuvant effect and is designed for the efficient delivery of antigens that can be easily tailored to the desired vaccine format^4–6^. The platform has been previously shown to induce potent, rapid, and persistent antibody and T cell responses against influenza antigens^12, 13^ as well as against the prototype Wuhan-Hu-1 receptor binding domain (RBD) of SARS-CoV-2^5^.

The antigenic unit of VB2129 contains the RBD of SARS-CoV-2, covering amino acid (aa) positions 319–542. The RBD domain was selected as an antigen because it drives the majority of neutralizing antibody responses against SARS-CoV-2, as demonstrated in COVID-19 patients^14–16^. With epitopes in RBD contributing to around 80% of the neutralization activity following infection or vaccination, vaccines based on the RBD alone can serve as effective boosters of immune responses primed by full-length spike proteins. The RBD variant encoded by VB2129 originated from lineage B.1.351 (Beta), which was first discovered in South Africa in late 2020 and was shown to be more resistant to neutralization by convalescent plasma and vaccine sera than the Wuhan prototype strain^17, 18^. The RBD of B.1.351 is characterized by three mutations, N501Y, E484K, and K417N, as compared to the RBD sequence of the original Wuhan strain (2020 prototype strain). These three mutations, also found in the Omicron VoC, have evolved to evade neutralization by RBD antibodies^19^. VB2129 has been used to generate broad protective immunity against SARS CoV-2 variants, particularly the newly-emerging VoC evading vaccine-induced immunity, such as Beta (B.1.351) or Omicron (B.1.1.529), which have been shown to greatly reduce the serum neutralizing antibody effect among individuals vaccinated with Wuhan spike-based vaccines^20, 21^.

In the present study, VB2129 induced strong binding and cross-neutralizing antibody responses in mice, accompanied by potent T cell responses against RBD epitopes. Candidate VB2129 described in this study is currently being investigated in a Phase 1 trial (NCT05069623).

## Results

### Design and in vitro characterization of VB2129 protein

**Figure 1:**
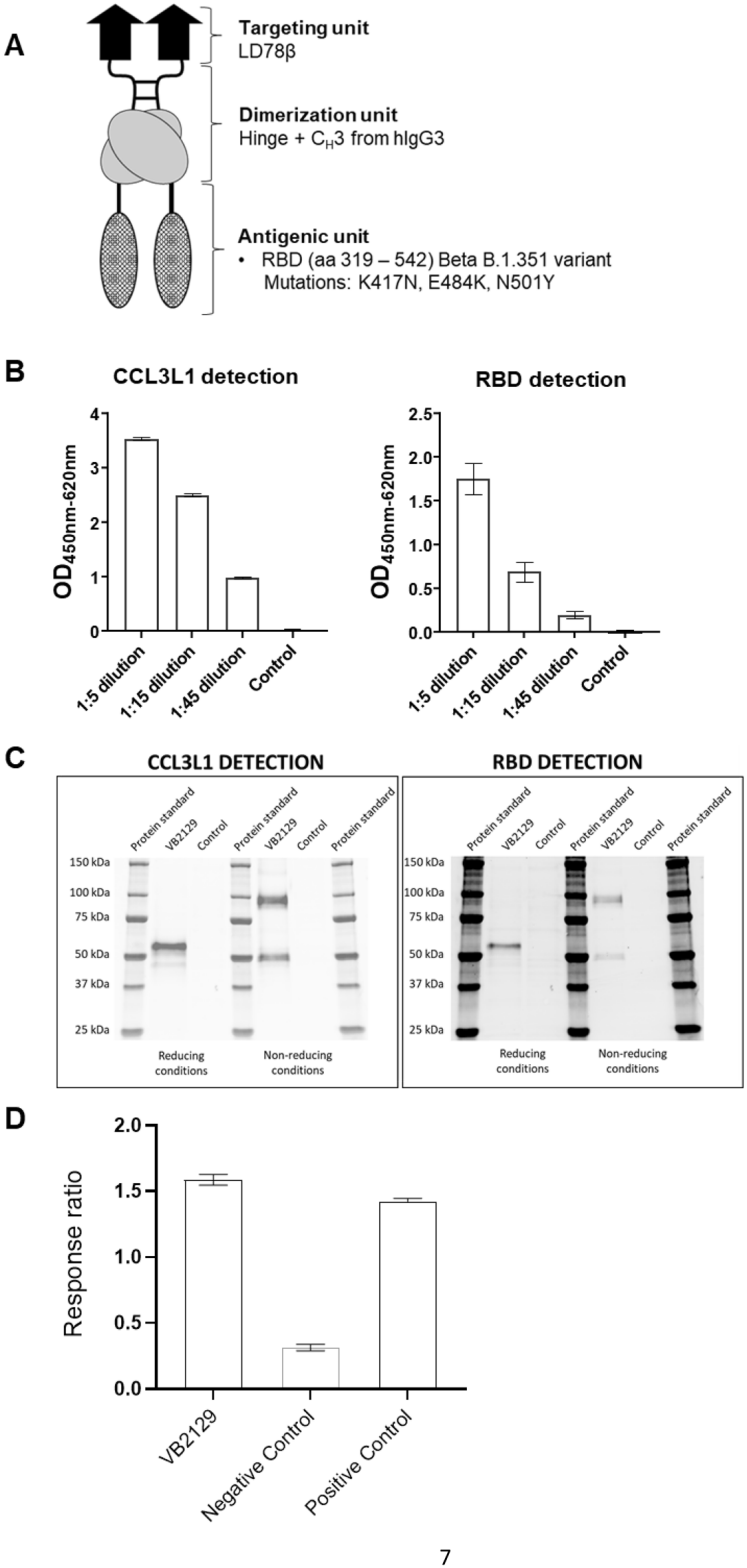
Design and characterization of the VB2129 vaccine candidate. (**A**) Schematic of the VB2129 homodimer. Each chain of the dimer contains (i) an N-terminal CCL3L1/LD78β targeting unit that is an isoform of the human CC chemokine macrophage inflammatory protein-1α (MIP-1α), (ii) a dimerization unit comprising a shortened IgG hinge and CH3 domain from human γ3 chains, and (iii) a C-terminal antigen unit linked to the dimerization unit. Antigen encoded by VB2129 was human-codon-optimized RBD from SARS-CoV-2 B.1.351; aa 319–542. (**B**) Secretion of VB2129 protein in mammalian cells. Expi293 cells were transiently transfected, and the supernatant was harvested at day 3 post-transfection and subjected to sandwich ELISA using antibodies directing human IgG CH3 domain for capturing; and human CCL3L1 or SARS-CoV-2 Wuhan-Hu-1 RBD for detection. (**C**) Western blot analysis of supernatant from Expi293 cells transfected with VB2129 at day 3 post-transfection. Reducing conditions visualize the monomeric form of the protein (approx. 55 kDa), and non-reducing conditions visualize the homodimeric form of the protein (approx. 95 kDa), using antibodies detecting human CCL3L1 or Wuhan-Hu-1 RBD. (**D**) Human CCR5 reporter assay in Tango hCCR5-bla U2OS cells using supernatant from VB2129-transfected cells or recombinant human CCL3L1 (positive control). The response ratio indicates the binding and activation of the hCCR5 receptor by CCL3L1. Supernatant from cells treated with transfection agent alone served as a negative control. Data presented in panels (**B**), (**C**) and (**D**) are from a single experiment representative of at least two experiments.

The antigenic component of the vaccine construct VB2129 was designed by including the B.1.351 mutations K417N, E484K, and N501Y (Fig. 1A) in a modified version of the Wuhan-Hu-1 RBD, which encompasses amino acids 319–542 of the spike protein^5^. Expression and secretion of dimeric vaccine proteins were evaluated following transfection of the mammalian suspension-adapted HEK293-based cell line Expi293. VB2129 was effectively expressed and translated into a Vaccibody vaccine protein, as verified by analysis of cell culture supernatant by sandwich ELISA (enzyme-linked immunosorbent assay) (Fig. 1B). Vaccine proteins were captured by specific antibodies against the dimerization unit hIgG C_H_3 and subsequently detected by antibodies against both the targeting unit CCL3L1 and the SARS-CoV-2 RBD antigenic domain of the protein (Fig. 1B). Western blot analysis of the cell culture supernatant confirmed the secretion of the full-length fusion protein and the formation of homodimers using antibodies detecting CCL3L1 or B.1.351 RBD (Fig. 1C). An hCCR5 reporter assay using Tango hCCR5-*bla* U2OS cells showed that CCL3L1 successfully bound to and activated hCCR5, as demonstrated by the difference in response ratios between cell culture supernatants from VB2129-transfected and negative control Expi293F cells. The results show that the CCL3L1 targeting unit in VB2129 is functional (Fig. 1D).

### Humoral immune responses induced in BALB/c mice vaccinated with VB2129

**Figure 2:**
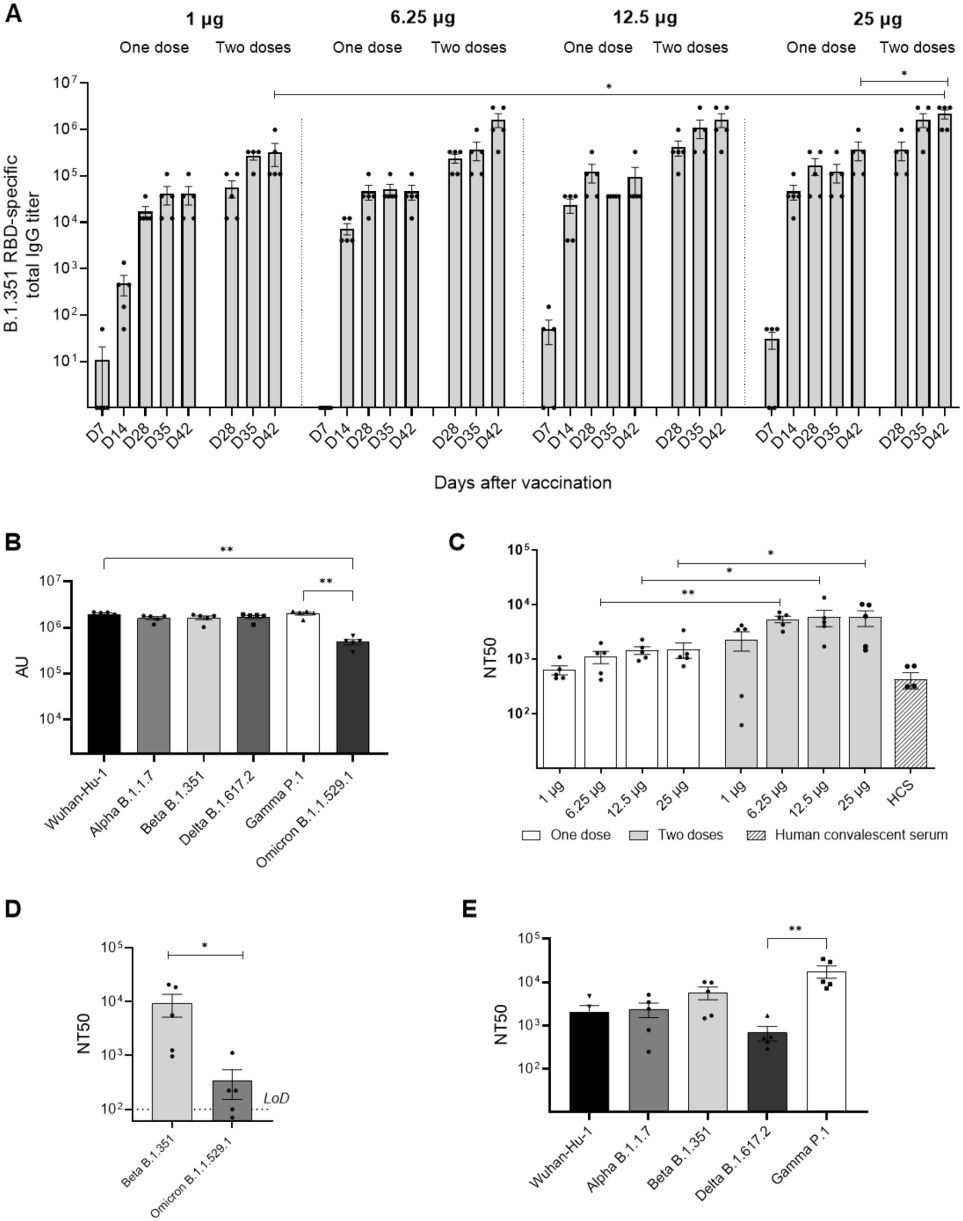
VB2129 vaccine evokes antibody responses in mice. BALB/c mice were vaccinated intramuscularly with one or two doses (day 0 or days 0 and 21) of 1, 6.25, 12.5 or 25 µg VB2129, and sera were collected at days 7, 14, 28, 35 and 42. (**A**) Time course of dose-dependent anti-B.1.351 RBD responses evoked by VB2129 as detected by ELISA. (**B**) Binding antibody responses in sera harvested at day 42 from BABL/c mice vaccinated with two doses of 25 µg VB2129. Sera were assessed for antibody binding to homologous B.1.351 RBD and heterologous Wuhan-Hu-1, B.1.1.7, 1.617.2, P.1 or B.1.1.529.1 RBD. (**C**) Dose-dependent neutralizing antibody responses in sera collected at day 42 post-prime vaccination and subjected to a homologous B.1.351 Spike pseudovirus neutralization assay. (**D**) Neutralizing antibody responses in sera harvested at day 42 from BABL/c mice vaccinated with two doses of 25 µg VB2129 and subjected to a homologous Beta B.1.351 and heterologous Omicron B.1.1.529.1 live virus neutralization assay. The dashed line indicates the limit of detection (LoD) at a starting dilution of 1:100. (**E**) Neutralizing antibody responses in sera harvested at day 42 from BABL/c mice vaccinated with two doses of 25 µg VB2129 and subjected to a homologous B.1.351 and heterologous Wuhan-Hu-1, B.1.1.7, B.1.617.2 or P.1 pseudovirus neutralization assay. The data are presented as average (per group) ± SEM (n = 5) with individual values. Statistically significant differences were determined using a two-tailed Mann–Whitney test for the comparison of two groups (panels **A**,**C**,**D**) or a Kruskal–Wallis test for the comparison of three or more groups (panels **B**,**E**) and are indicated as follows: *p < 0.05; **p < 0.01. The data in panels (**B**), (**C**) and (**E**) are presented after background (PBS group) subtraction.

Antigen-specific antibody responses induced by VB2129 were assessed in serum samples from vaccinated BALB/c mice. VB2129 induced strong anti-B.1.351 RBD antibody responses at day 7 post-vaccination with a single dose of 12.5 or 25 µg plasmid DNA (Fig. 2A). Antibody levels reached titers of 10^4^ on day 14 for all doses tested. A second vaccination on day 21 enhanced the antibody responses at all dose levels tested, demonstrating a promising effect of booster vaccination (Fig. 2A). Peak responses on day 42 for the two-dose regimen reached titers of 10^6^ for doses of 6.25, 12.5, and 25 µg (Fig. 2A). Serum samples from mice vaccinated with two doses of 25 µg VB2129 harvested at day 42 post-prime vaccination were evaluated for antibody binding to heterologous RBD proteins of SARS-CoV-2 (Table 1). The results confirmed that VB2129 induces strong antibody responses with comparable binding to Wuhan-Hu-1, B.1.1.7, B.161.2, and P.1 RBD (titers > 10^6^) (Fig. 2B). In addition, strong but lower levels of binding antibodies were detected against B.1.1.529.1 (titers > 10^5^).

**Table 1:** Amino acid differences in the RBD region between SARS-CoV-2 RBD antigens or strains tested in binding and neutralizing antibody assays.

Strain	RBD mutations
Alpha B.1.1.7	N501Y
Beta B.1.351	K417N, E484K, N501Y
Delta B.1.617.2	T478K, P681R, L452R
Gamma P.1	417T, E484K, N501Y

Sera from mice vaccinated with one or two doses of 1, 6.25, 12.5, or 25 µg VB2129 and harvested at day 42 post-prime vaccination were assessed in a B.1.351 pseudovirus neutralization assay. Strong and dose-dependent neutralizing activity was detected following a single vaccination at all dose levels tested, and neutralizing antibody responses were further increased by the second dose of VB2129 (Fig. 2C). All regimens reached higher titers than those detected in human convalescent serum, even at the lowest doses tested (Fig. 2C). Sera from mice vaccinated with two doses (25 µg) and harvested on day 42 were further investigated for an ability to neutralize homologous Beta B.1.351 and Omicron B.1.1.529.1 in live virus neutralization assays. The analysis showed high titers of neutralizing antibodies against the homologous strain and, importantly, noticeable neutralizing activity against Omicron BA.1 VoC in four out of five sera tested (Fig. 2D). Next, sera were tested for neutralizing activity against pseudoviruses expressing spike proteins for common variants of concern: Wuhan-Hu-1, B.1.1.7, B.1.617.2, P.1, and B.161.2. VB2129-induced antibodies neutralized all tested pseudovirus variants, with the highest activity observed against P.1 RBD-expressing pseudovirus (titers > 10^4^). Notably, P.1 (Gamma) VoC shares two of the three mutations in RBD with B.1.351^22^. The lowest neutralizing activity was observed against B.1.617.2 RBD-expressing pseudovirus (titers < 10^3^), which can be explained by the fact that there is only one shared mutation between B.1.351 and B.1.617.2 RBDs (K417N)^23^ (Fig. 2E). Interestingly, binding antibody responses did not correlate with neutralizing activity against Delta B.1.617.2 pseudovirus, which was lower compared to other pseudoviruses tested (Fig. 2B,E). This can be explained by the fact that the Delta B.1.617.2 variant was shown to spread by escaping from antibodies that target not only RBD epitopes, but also non-RBD epitopes within the Spike protein^24^.

### T cell responses in BALB/c mice to RBD variant protein epitopes

**Figure 3:**
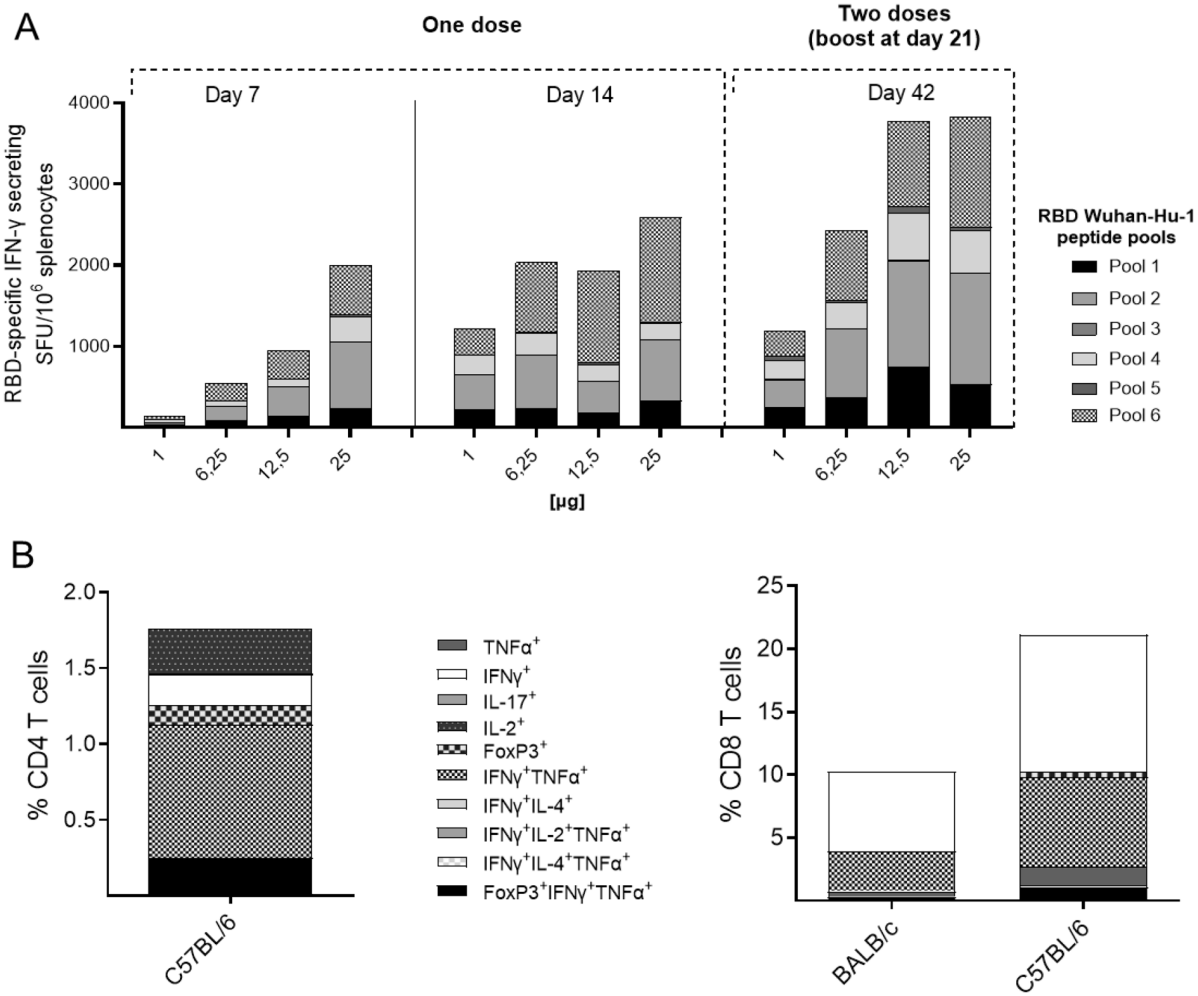
VB2129 vaccine evokes T cell immunity in mice. BALB/c mice were vaccinated with one or two doses of 1, 6.25, 12.5 or 25 µg VB2129 and the induced T cell responses were recalled in splenocytes from vaccinated mice at day 7 or 14 (single dose vaccination) or day 42 (two-dose vaccination) with peptide pools composed of peptides covering the Wuhan-Hu-1 RBD sequence and analyzed by IFN-γ ELISpot. The data are presented as a total response to the individual peptide pools (n = 5, pooled spleens). (**A**) BALB/c and C57BL/6 mice were vaccinated with two doses of 25 µg. Splenocytes harvested at day 42 and stimulated with peptide pools of Wuhan-Hu-1 RBD were assessed by cytokine profiling and analyzed by flow cytometry (**B**) The data are presented as a total response representing induced cytokine profiles (n = 5, pooled spleens).

Next, the ability of VB2129 to induce T cell immunity against RBD-variant protein epitopes was investigated. A single vaccine dose induced strong dose-dependent T cell responses recalled with specific Wuhan-Hu-1 RBD peptide pools (Fig. 3A). Specific T cell responses were observed as early as day 7 for doses of 6.25, 12.5, and 25 µg and further increased at day 14. T cell responses were also observed on day 14 at the lowest dose of 1 µg. A second dose of VB2129 administered on day 21 further increased specific T cell responses, as assessed on day 42 for all dose levels (Fig. 3A). The T cell responses induced by two doses of VB2129 were recalled with both Wuhan-Hu-1 and B.1.351 RBD-specific peptide pools and showed similar numbers of IFN-γ-secreting splenocytes and patterns of response to individual peptides. The results suggest that mutations in the RBD of the B.1.351 variant do not substantially alter RBD-specific T cell immunity in the mouse model tested (Fig. S1).

Functional profiling of T cells was performed by flow cytometry using splenocytes from BALB/c or C57BL/6 mice vaccinated with 25 µg VB2129 at two different time points and harvested at day 42 post-prime vaccination. RBD-specific CD4^+^ T cells were undetectable in BALB/c mice, whereas CD4^+^ T cell responses in C57BL/6 mice were dominated by IFN-γ^+^ TNF-α^+^ (approx. 0.87%) and IFN-γ (approx. 0.2%) positive cells, indicating induction of a typical Th1 profile (Fig. 3B). In contrast to CD4^+^ T cell responses, the CD8^+^ T cell responses were strong in both mouse models (approx. 10% in BALB/c mice; > 20% in C57BL/6 mice), and cells exhibited a typical proinflammatory profile with production of IFN-γ or a combination of IFN-γ^+^ and TNF-α^+^ (Fig. 3B).

## Discussion

The currently approved vaccines for COVID-19 are safe and effective in preventing infection and severe disease caused by the original Wuhan-1 strain; however, emerging VoC can escape neutralizing antibodies and, to a lesser degree, escape cell-mediated immunity^25^. This reduces the effectiveness of the vaccines against some VoC, with 22 to 60% lower protection reported against the Beta strain B.1.351 infections, and even lower against Omicron VoC^26^. Preclinical challenge studies in mice vaccinated with parental candidate mRNA-1273 (Wuhan Spike variant mRNA vaccine, Moderna, U.S.) have shown that a lower vaccine dose was less effective against the B.1.351 strain, while the modified mRNA-1273.351 vaccine was generally protective^27^.

This study presents a COVID-19 vaccine candidate, VB2129, designed to address a need for vaccines to induce potent and persistent antibody responses with neutralizing activity against emerging strains, accompanied by T cell immunity. To ensure robust antibody responses, one major objective in vaccine development against infectious diseases is the enhancement of the germinal center (GC) reaction, where B cells with high-affinity BCRs are selected for survival and develop into long-lived memory B cells and antibody-secreting plasma cells. It has previously been suggested and recently demonstrated^11^ that APC-targeted molecules^10, 28, 29^ facilitate the formation of APC-B cell synapses that augment the GC reaction, resulting in rapid and increased antibody responses with enhanced avidity. The strong and persistent RBD-specific antibody responses elicited by the VB2129 candidate may be conferred by the homodimeric vaccine design in combination with APC-targeting, allowing bivalent display of RBD antigen thereby facilitating cross-linking of BCRs in an APC-B cell synapse to amplify GC B cells and long-lived plasma cells^3^.

One benefit of the VB2129 vaccine candidate is its safety. The safety of DNA vaccine formats presented in this study encoding a chimeric protein has been evaluated in clinical programs for therapeutic cancer vaccines, either carrying HPV16 E6/E7 antigens and targeting intraepithelial neoplasia (NCT02529930) or recurrent cervical cancer (NCT04405349) or as a fully personalized cancer neoantigen vaccine in patients with locally advanced or metastatic cancers (NCT03548467), and no substantial safety concerns have emerged to date^30^. Furthermore, more than 15 DNA vaccine candidates against COVID-19 have been evaluated in clinical trials (*COVID-19 vaccine tracker and landscape*, WHO), including ZyCoV-D, and have demonstrated desirable safety profile and tolerability. The ZyCoV-D vaccine was immunogenic in humans using intradermal jet delivery, showed a protective efficacy of 66.6% and is the world’s first approved DNA vaccine for preventing infectious disease in humans^31^.

Neutralizing antibody activity against SARS-CoV-2 correlates with protection against infection, as demonstrated in animal studies^32^, natural infections^33^, and large-scale Phase 3 efficacy studies with several vaccine candidates^34, 35^. Here, we demonstrated that the chimeric DNA vaccine constructs VB2129 encoding RBD of the B.1.351 variant induced rapid and robust cross-binding and cross-neutralizing antibody responses in mice, suggesting vaccine efficacy against different VoC, including Omicron. The Omicron variant is associated with immune escape from neutralizing antibodies induced by prototype spike-based vaccines or infections^36, 37^. Vaccination with two doses of ChAdOx1 nCoV-19 or BNT162b2 was ineffective in protecting against symptomatic disease caused by infection with the Omicron strain. Although both homologous and heterologous boosters with approved mRNA vaccines after a primary two-dose course increased protection, the effectiveness declined over time^38^. Booster vaccination that confers durable immunity against this VoC remains crucial for mitigating the ongoing pandemic. Here, we showed that vaccination with Beta RBD induced cross-reactive neutralizing antibodies against a live virus Omicron BA.1 isolate (Fig. 2D). We hypothesize that using Beta RBD as a heterologous booster may stimulate further antibody diversity and cross-neutralizing specificities, as recently clinically demonstrated for a Beta spike-based mRNA vaccine^39^.

While most studies investigating immune responses to COVID-19 vaccines or during SARS-CoV-2 natural infections have focused on neutralizing antibodies, cellular immunity is likely to play a critical role in protection against diseases caused by SARS-CoV-2^2^. We showed that the VB2129 candidate induced strong T cell immunity against overlapping epitopes spanning the RBD as early as day 7 after vaccination and that the responses were dominated by CD8^+^ T cells of the proinflammatory profile. As spike mRNA vaccines against SARS-CoV-2 can protect against severe disease around 10 days after vaccination^40, 41^, in contrast to neutralizing antibodies that are detectable after day 21^42, 43^, CD8^+^ T cells have been suggested as mediators of protection at this early stage^44^. The potential key role of T cells in protection against severe disease is supported by evidence of cellular-mediated cross-protection against VoC, which may evade vaccine-induced antibody responses^45^.

The SARS-CoV-2 VoC reported to date are known to be more transmissible, with some causing more severe disease (e.g., Delta) and others severely impacting the effectiveness of approved vaccines (e.g., Beta and Omicron BA.1) by immune evasion^46^. Thus, the present VB2129 vaccine candidate could be a booster vaccine for broader protection against current and future SARS-CoV-2 variants. Importantly, as described in this study, a safe and highly versatile vaccine platform can be designed for emerging virus variants to induce broad neutralizing antibodies and strong T cell responses, allowing for a prompt response to the global need for vaccines against emerging infectious diseases.

## Materials and methods

### Plasmid construction

The antigenic unit of VB2129, B.1.351 RBD, was created by single amino acid mutagenesis (K417N, E484K, and N501Y) of the synthesized Wuhan RBD^5^ and cloned into a pUMVC4a VB10 master plasmid using *Sfi*I-*Sfi*I restriction enzyme sites as described previously^5^. The resulting construct encoded homodimeric proteins with CCL3L targeting units^4^ and RBD antigenic units, connected via a homodimerization unit consisting of exons from hinges h1 and h4 and C_H_3 of human IgG3 (Fig. 1A). The sequences encoding the protein constructs were codon-optimized. The designed plasmids were constructed, cloned, and produced by GenScript (Piscataway, NJ).

### Transient transfection in Expi293 cells

VB2129 secretion was assessed by transient transfection of Expi293F suspension cells (Thermo Fisher Scientific, Waltham, MA). Expi293F cells were diluted to 2.8 × 10^6^/mL and incubated overnight in a CO_2_ incubator (8% CO_2_, 37 °C) with orbital shaking (125 rpm, 19 mm diameter). 2.0 × 10^6^ cells/well) were seeded in deep 96-well plates and transfected with 0.64 µg pDNA complexed with 2 µL ExpiFectamine 293 Reagent (Thermo Fisher) according to the manufacturer’s instructions. Mock transfection with ExpiFectamine 293 reagent alone served as a control. The cells were incubated for three days in a CO_2_ incubator (8% CO_2_, 37 °C) with orbital shaking (900 rpm, 3 mm diameter). Supernatants were harvested by centrifugation at 300×*g* for 5 min and further cleared at 4000×*g* for 15 min at 4 °C. The supernatants were subjected to sandwich ELISA, Western blotting, and a human (h)CCR5 reporter assay.

### ELISA assay for detection of secreted VB2129 protein

ELISA plates (Nunc MaxiSorp, Thermo Fisher) were coated with anti-IgG CH3 antibody (Bio-Rad, Hercules, CA; 1:500) overnight at 4 °C, followed by blocking with 4% bovine serum albumin (BSA) in PBS for 1 h at RT (room temperature). The supernatants were added to the wells and incubated for 1 h at 37 °C. For detection of CCL3L1, plates were washed and incubated with biotinylated anti-human MIP-1α (R&D Systems, Minneapolis, MN; 1:1,000), followed by streptavidin-peroxidase polymer (Sigma-Aldrich, St Louis, MO; 1:3,000). For detection of RBD, plates were incubated with rabbit anti-SARS-CoV-2 (2019-nCoV) Spike RBD Antibody (Sino Biological, Beijing, China; 1:1,000) followed by HRP-conjugated anti-rabbit IgG antibody (Invitrogen, Waltham, MA; 1:5,000). All incubations were performed for 1 h at 37 °C. The plates were then washed and TMB (3,3ʹ,5,5ʹ-tetramethylbenzidine) solution was added (Merck, Darmstadt, Germany). Color development was stopped after 10 min by adding 1 M HCl. Optical density was measured at 450 nm using a Tecan SPARK plate reader (Bergman Diagnostika AS, Kjeller, Norway).

### Western blotting

For western blot analysis, clarified Expi293 cell supernatants were mixed with 4 × Laemmli buffer (Bio-Rad) and dithiothreitol (reduced samples, 50 mM final concentration, Cayman Chemicals, Ann Arbor, MI) or water (non-reduced samples). Samples were heated at 70 °C for 10 min prior to loading on a 4–20% Criterion TGX Stain-Free Protein Gel (Bio-Rad). The Precision Plus Protein Standard (Bio-Rad) was used to determine the molecular weights (detection in the Dylight 650 channel). Following gel electrophoresis (200 V, 42 min), the proteins were transferred to low fluorescence polyvinylidene difluoride (PVDF) membranes (Bio-Rad) using the Trans-Blot Turbo system (7 min, 2.5 A) and Trans-Blot Turbo Transfer Buffer (Bio-Rad). Stain-Free total protein visualization (ChemiDoc MP, Bio-Rad) was used to verify equal loading and transfer of the samples and controls. The membranes were blocked with EveryBlot buffer (EB, Bio-Rad) for 5 min at RT. Proteins were detected with goat anti-MIP-1α antibody (R&D Systems, 1:1000 in EB, 4 °C overnight) to detect CCL3L1. Membranes were washed with Tris-Buffered Saline, 0.05% Tween-20 Detergent (TBST, Cell Signaling Technology, Sigma) and incubated with Dylight 800-conjugated secondary anti-goat antibody (Thermo Fisher, 1:5000 in EB with 0.01% SDS (sodium dodecyl sulfate), VWR) for 1 h at RT. The membranes were washed with TBST and dried. For reprobing, the membranes were blocked, probed, washed, and dried as described above using rabbit anti-SARS-CoV-2 (2019-nCoV) Spike RBD Antibody (Sino Biological, 1:1000 in EB, 4 °C, overnight) and Dylight 650-conjugated secondary anti-rabbit antibody (Thermo Fisher, 1:5000 in EB with 0.01% SDS, 1 h, RT). Images were acquired with the ChemiDoc MP Imaging System (Bio-Rad) and analyzed with ImageLab 6.1 (Bio-Rad).

### hCCR5 reporter assay

An hCCR5 cellular reporter assay was carried out using Tango hCCR5-*bla* U2OS cells (Invitrogen). Activation of hCCR5 in these cells induces an intracellular signaling cascade leading to the expression of β-lactamase (*bla*), which can be measured by fluorescent detection of the *bla* substrate, CCF4-AM. In short, hCCR5-*bla* U2OS cells were cultured in McCoy’s 5A Medium, supplemented with 10% dialyzed FBS (Gibco, Thermo Fisher), 1% penicillin–streptomycin, 0.1 mM non-essential amino acids (Gibco), 25 mM HEPES (Gibco), 1 mM sodium pyruvate (Gibco), 200 µg/mL Zeocin (Gibco), 100 µg/mL Geneticin (Gibco), and 50 µg/mL Hygromycin B (Gibco). For the hCCR5 reporter assay, cells were collected using Cellstripper (Corning, Corning, NY; 8 min, 37 °C, 5% CO_2_) and centrifuged (300 × g, 5 min, RT) before they were resuspended in Expi293 expression medium (Gibco). Next, 40,000 cells (50 µL cell suspension) were seeded in each well of black, optical-bottom 96-well plates (Corning), and 100 µL supernatant from Expi293F cells transiently transfected with VB2129 or ExpiFectamine (mock) was added to each well in triplicate. Recombinant human CCL3L1 (R&D Systems) was used as the positive control. Wells with Expi293 expression medium were included as background (no cell) controls. Plates were incubated for 16 h (37 °C, 5% CO_2_), before 30 µL of a CCF4-AM *bla* substrate mix (Invitrogen) was added (prepared in-house according to the manufacturers’ protocol), and the plates were incubated for another 2 h (RT, in the dark). The samples were analyzed using a Spark microplate reader (Tecan) set to optimal fluorescence gain. Two fluorescent scans were performed (Scan #1: excitation, 409 nm; emission, 460 nm; Scan #2: excitation, 409 nm; emission, 530 nm). The values from the no-cell background wells were subtracted from the sample wells, and the response ratio between the relative fluorescence unit (RFU) values from Scan #1 (detection of the *bla* substrate product) and Scan #2 (detection of the *bla* substrate) was obtained. The experiments were performed in triplicate.

### Immunogenicity studies

#### Immunization of animals

All animal experiments are described in accordance with ARRIVE guidelines recommendations. All procedures were reviewed and approved by Norwegian Animal Research Authority (Mattilsynet, Norway) and were carried out in accordance with the recommendations from the Guide for the Care and Use of Laboratory Animals of the Norwegian National Institute of Health (FOTS ID 25797). Female BALB/c and C57BL/6 mice were obtained from Janvier Laboratories (Le Genest-Saint-Isle, France) and randomly divided into experimental groups. All animals were housed in the animal facility at the Domus Medica, Department of Comparative Medicine, University of Oslo (Oslo, Norway). BALB/c mice were given either one dose (day 0) or two doses (days 0 and 21) of VB2129 vaccine administrated to each tibialis anterior (TA) muscle by needle injection followed by AgilePulse in vivo electroporation (BTX, Holliston, MA). The vaccine doses were 1, 6.25, 12.5, and 25 µg. Blood samples were collected on days 7, 14, 28, and 35. Spleens were collected on days 7, 14, and 42. C57BL/6 mice were vaccinated with two doses of 25 µg VB2129, and spleens were harvested on day 42 for flow cytometry analysis.

#### Detection of RBD IgG in serum samples

The humoral immune responses specific for homologous B.1.351 RBD were evaluated in sera from mice vaccinated with one or two doses of 1, 6.25, 12.5, or 25 µg VB2129 using an ELISA assay detecting total IgG at days 7, 14, 28, 35, and 42 post prime vaccination. Briefly, ELISA plates were coated with 2.5 µg/mL B.1.351 RBD protein overnight at 4 °C, followed by blocking with 4% BSA in PBS for 1 h at RT. Plates were then incubated with serial dilutions of sera, washed, and further incubated with anti-mouse IgG-HRP (horseradish peroxidase) antibody (Southern Biotech, Birmingham, AL) for 1 h at 37 °C. Plates were developed using TMB substrate (Merck), and color development was stopped after 10 min by the addition of 1 M HCl. Plates were read at 450 nm using Multiscan GO (Thermo Fischer). Binding antibody endpoint titers were calculated as the reciprocal of the highest dilution resulting in a signal above cutoff.

The levels of IgG antibodies binding RBD variants, Wuhan-Hu-1, B.1.1.7 Alpha, B.1.351 Beta, B.1.617 Delta, P.1 Gamma, and B.1.1.529.1 Omicron BA.1, were quantified in sera from mice vaccinated with two doses of 25 µg VB2129 at day 42 post prime vaccination, using V-PLEX SARS-CoV-2 Panel 22 (Mouse IgG) Kit (Mesoscale, Rockville, MD) according to the manufacturer’s protocol. A mixture of sera from 8 mice was used as an interplate control. The plates were read on a MESO Quickplex SQ120, and the results were analyzed using the associated software (Discovery Workbench). The raw data were plotted as arbitrary units (AU), as no standard curve was available.

#### Evaluation of functional antibody activity by neutralization assay

A pseudovirus neutralization assay was performed in Nexelis (a Q^2^ Solutions Company, Laval, Canada) using serum samples from VB2129-vaccinated mice and Vero E6 cells expressing the ACE-2 receptor. The WHO International Standard for anti-SARS-CoV-2 immunoglobulin, human (NIBSC code: 20/136) was used as a positive control. Cells were seeded in 96-well plates (2 × 10^5^ cells/well) and incubated overnight at 37 °C with 5% CO_2_. SARS-CoV-2 pseudoviruses Wuhan-Hu-1, B.1.351 Beta, B.1.617.3 Delta, and P.1 Gamma were added at predetermined concentrations (TU/mL) to serially diluted serum samples and incubated for 1 h at 37 °C with 5% CO_2_. The mixture was added to Vero E6 cells and incubated for 18–20 h at 37 °C with 5% CO_2_. Following incubation and removal of the media, ONE-Glo EX Luciferase Assay Substrate (Promega, Madison, WI) was added to the cells, which were then incubated for 3 min at room temperature with shaking. Luminescence was measured using a SpectraMax ID3 microplate reader and SoftMax Pro v7.01 (Molecular Devices, San Jose, CA). Luminescence results for each dilution were used to generate a titration curve using a 4-parameter logistic regression. The titer was defined as the reciprocal dilution of the sample for which the luminescence was equal to a predetermined cut-point of 50, corresponding to 50% neutralization (NT50). This cut-point was established using linear regression with 50% flanking points.

#### Live SARS-CoV-2 neutralization assay

Serum samples from VB2129-vaccinated mice from day 42, following two doses of 25 µg vaccine, were used to assess the capacity of the antibody to neutralize full-length SARS-CoV-2 B.1.351 351 (Human nCoV19 isolate/England ex-SA/HCM002/2021, European Virus Archive—Global) or B.1.1.529.1 (Isolated at the Department of Infectious Diseases at Aarhus University Hospital). Both B.1.351 and B.1.1.529.1 strains were sequenced, and the sequences were verified using Pangolin online free software (https://pangolin.cog-uk.io). Sera were heat-inactivated for 45 min at 55 °C prior to assay Eight fivefold dilutions of sera were plated in a 96-well plate and incubated with one of the SARS-CoV-2 variants for 1 h at 37 °C in 5% CO_2_ followed by addition of 20,000 Vero/hTRMPSS2 cells per well. SARS-CoV-2 was used at an MOI of 0.0005. Cells, viruses, and sera were incubated in Dulbecco’s modified Eagle medium with the addition of 1% penicillin/streptomycin and 2% FBS for 72 h at 37 °C at 5% CO_2_, after which each well was scored in a binary fashion as infected or neutralized. NT50 values were calculated using the Reed-Muench method.

#### IFN-γ ELISpot assay

To detect RBD-specific T cell responses, splenocytes from vaccinated mice were analyzed using the IFN-γ ELISpot assay. Briefly, BALB/c mice were sacrificed at days 7, 14 or 42 post-vaccination, and the spleens were harvested aseptically. The spleens were homogenized, and single-cell suspensions were incubated with 1 × ACK buffer to remove erythrocytes, washed, and resuspended to a cell concentration of 6 × 10^6^ cells. The cells were plated in triplicate and stimulated with 2 µg/ml of Wuhan-Hu-1 or B.1.351 RBD peptide pools (6 pools consisting of 10–11 15mers overlapping with 12 aa) for 24 h. Cells without peptide stimulation were used as negative controls. Stimulated splenocytes were analyzed for IFN-γ response using a mouse IFN-γ ELISpot Plus kit (Mabtech AB, Stockholm, Sweden). Spot-forming units (SFU) were measured using an IRIS ELISpot reader and APEX™ software (Mabtech AB). Results are shown as the mean number of IFN-γ + spots/10^6^ splenocytes with a subtracted background.

#### Flow cytometry analysis

Splenocytes from BALB/c or C57BL/6 mice vaccinated with two doses of 25 µg VB2129 (days 0 and 21) and harvested at day 42 after prime vaccination were prepared as described for IFN-γ ELISpot assay. Cells (2.0 × 10^6^) were stimulated for 6 h with 6 µg/ml Wuhan-Hu-1 RBD peptide pool; 1 × monensin and 1 × brefeldin were added to the wells at 1 h post incubation. Following stimulation, cells were harvested and centrifuged twice with PBS to remove the medium. Cells were incubated with a fixable viability dye (eFluor780, Thermo Fisher) in the dark for 10 min at RT. Cells were further stained with extracellular antibodies (anti-CD3, anti-CD4, anti-CD8, and γδTCR), fixed, permeabilized, and stained for detection of cytokines (anti-TNF-α, anti-IFN-γ, anti-IL-2, anti-IL-4 anti-IL-17 antibodies) and a transcription factor (anti-FoxP3 antibody). The stained cells were run in BD FACSymphony A5 (BD Biosciences, San Jose, CA) and analyzed using FlowJo software (Becton, Dickinson & Co., Franklin Lakes, NJ) with the Boolean gating algorithm. Signal obtained for the PBS negative control mice was deducted from the signal obtained for the VB2129-vaccinated mice for each cytokine.

### Supplementary Information

Supplementary Figure 1.

## Data Availability

The data supporting the findings of this study are available from the corresponding author, KK, upon reasonable request.
