# Supplementary material for: A novel SARS-CoV-2 Beta RBD DNA vaccine directly targeted to antigen-presenting cells induces strong humoral and T cell responses
Source: Sci Rep. 2023 Nov 2;13:18902. doi: 10.1038/s41598-023-46223-8 (PMC10622562; doi:10.1038/s41598-023-46223-8)

**Supplementary figure**


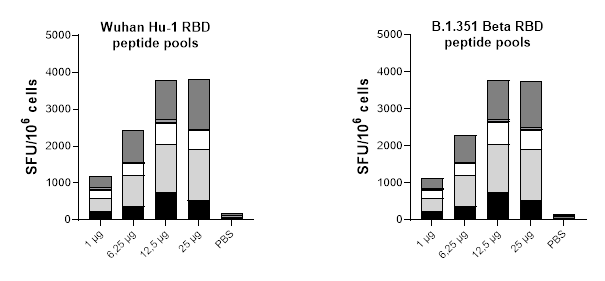
**Figure S1. VB2129-induced T cell immunity recalled by peptide pools covering Wuhan-Hu-1 or B.1.351 Beta RBD.** BALB/c mice were vaccinated with two doses of 1, 6.25, 12.5 or 25 µg VB2129. The induced T cell responses were recalled at day 42 with peptide pools comprising peptides covering the Wuhan-Hu-1 or B.1.351 Beta RBD sequence and analyzed by IFN-γ ELISpot. Data are presented as total cell responses to individual peptide pools (n=5, pooled spleens).

**Supplementary uncropped gel images presented on Figure 1C**


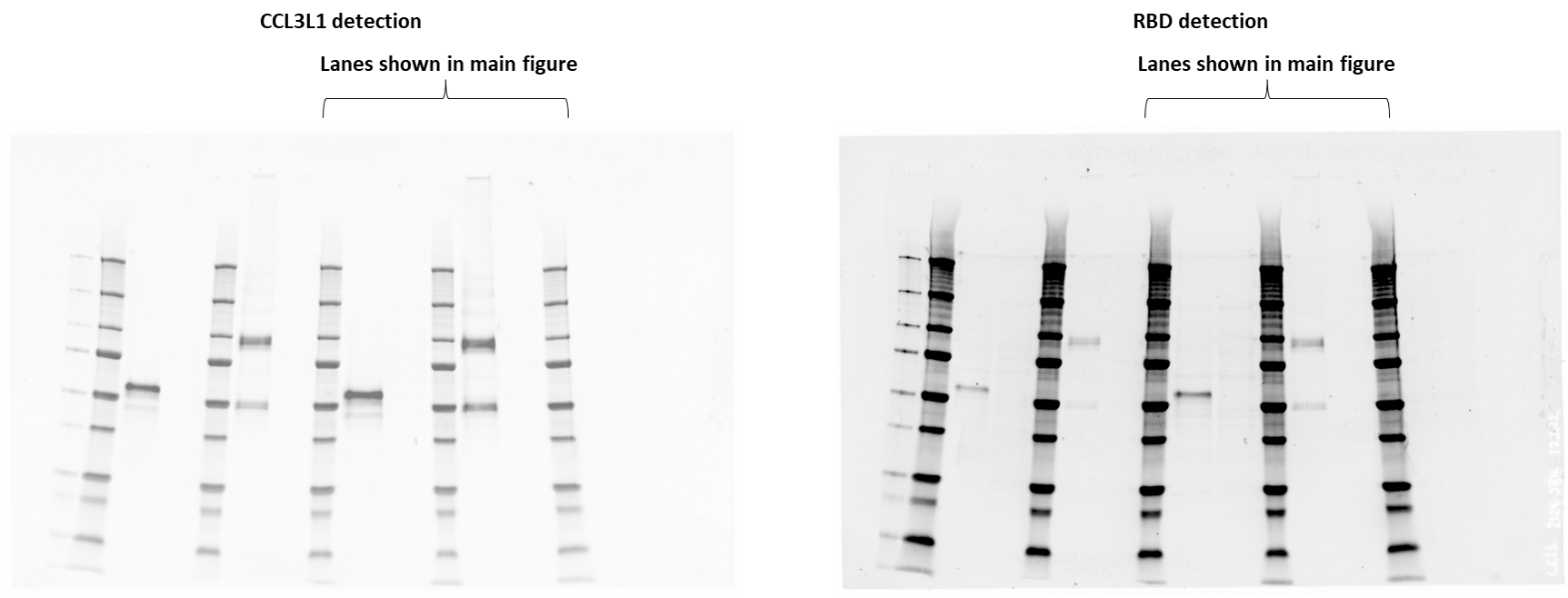

Supplement: Supplementary file 1 — Supplementary Figure 1. [file 41598_2023_46223_MOESM1_ESM.docx]
